# Effects of Lentivirus-Mediated C3 Expression on Trabecular Meshwork Cells and Intraocular Pressure

**DOI:** 10.1167/iovs.18-24978

**Published:** 2018-10

**Authors:** Junka Tan, Nin Fan, Ningl Wang, BingKa Feng, Min Yang, Gu Liu, Yu Wang, Xianju Zhu, Paul L. Kaufman, Iok-Ho Pang, Xuyan Liu

**Affiliations:** 1 Shenzhen Key Laboratory of Ophthalmology, Shenzhen Eye Hospital, School of Optometry, Shenzhen University, Shenzhen, China; 2 Tongren Eye Center, Beijing Tongren Hospital, Capital Medical University, Beijing, China; 3 Institute of Laboratory Animal Sciences, Sichuan Academy of Medical Sciences and Sichuan Provincial Hospital, Chengdu, Sichuan, China; 4 Department of Ophthalmology and Visual Sciences, University of Wisconsin-Madison, Madison, Wisconsin, United States; 5 Department of Pharmaceutical Sciences and North Texas Eye Research Institute, University of North Texas Health Sciences Center, Fort Worth, Texas, United States

**Keywords:** lentivirus, C3 transferase, trabecular meshwork, rat, intraocular pressure

## Abstract

**Purpose:**

We evaluated the effects of lentivirus-mediated exoenzyme C3 transferase (C3) expression on cultured primary human trabecular meshwork (HTM) cells in vitro, and on rat intraocular pressure (IOP).

**Methods:**

HTM cells were cultured and treated with lentivirus vectors expressing either green fluorescent protein (GFP) only (LV-GFP) or GFP and C3 together (LV-C3-GFP). Changes in cell morphology and actin stress fibers were assessed. The vectors were also injected into the anterior chamber of rats, and GFP expression was visualized by a Micron III Retinal Imaging Microscope in vivo and a fluorescence microscope ex vivo. Changes in rat IOP were monitored by using a rebound tonometer and the eyes were evaluated by slit lamp.

**Results:**

LV-mediated C3 expression induced morphologic changes in HTM cells. The cells became retracted and rounded. GFP expression in the anterior chamber angle of rats was observed in vivo from 8 days to 48 days after injection of LV-C3-GFP or LV-GFP. IOP was significantly decreased in the LV-C3-GFP group starting 3 days post injection, and lasting for at least 40 days, when compared to either the contralateral control eyes (the LV-GFP group) or the ipsilateral baseline before injection (*P* < 0.05). No obvious inflammatory signs were observed in either the LV-C3-GFP or LV-GFP groups.

**Conclusions:**

LV-mediated C3 expression induced changes in morphology of cultured HTM cells. Intracameral injection of LV-C3-GFP lowered rat IOP for at least 40 days. No significant inflammatory reactions were observed in either the LV-C3-GFP or LV-GFP groups. This study supports the possible use of *C3* gene therapy for the treatment of glaucoma.

The actin cytoskeleton and its associated cellular interactions in the trabecular meshwork (TM) and juxtacanalicular tissue (JCT) contribute to aqueous outflow resistance. Direct or indirect actin-disruptive agents, such as the serine-threonine kinase inhibitor H-7 and latrunculins A and B, can disrupt the actin cytoskeleton and alter cell morphology and architecture in the TM and JCT, and thereby change the overall TM and Schlemm's canal geometry and decrease outflow resistance. Rho-mediated signaling pathways regulate the assembly and contractility of the actomyosin network. Rho-associated protein kinase (ROCK) is one of the major downstream effectors of Rho GTPases, and the inhibition of ROCK lowers IOP of rats, rabbits, monkeys, and human patients with primary open-angle glaucoma.^1–4^ Exoenzyme C3 transferase (C3) is isolated from *Clostridium botulinum* and specifically inactivates Rho by ADP-ribosylation.^5^ We hypothesized that C3 has effects on aqueous outflow and IOP similar to ROCK inhibitors. Our previous work^6^ has shown that adenovirus-mediated C3 expression can significantly induce morphologic changes in human trabecular meshwork (HTM) cells and increase outflow facility in organ-cultured monkey anterior segments. In the current study, we report that lentivirus-mediated C3 delivery altered the cytoskeleton and its associated adhesions of cultured HTM cells and lowered intraocular pressure (IOP) in living rat eyes. Our findings support the possible use of *C3* gene therapy for the treatment of glaucoma.

## Materials and Methods

### Viral Vectors

**Figure 1:**
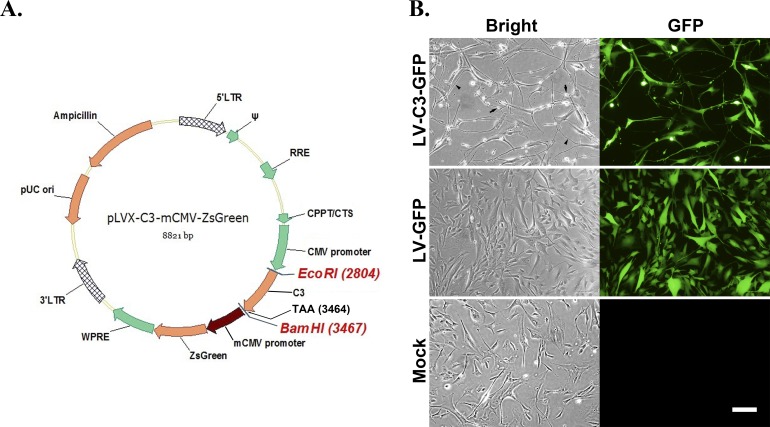
Morphology of cultured HTM cells after transduction with lentivirus vectors. (A) Lentiviral construct. (B) Cells in 24-well plate transduced with LV-C3-GFP and LV-GFP, respectively, both at MOI of 400. The LV-C3-GFP–transduced cells appeared to be either rounded up (arrows) or elongated (arrowheads) for at least 144 hours (end of observation) when compared to LV-GFP–transduced cells and medium-only–treated control (mock). Scale bar: 100 μm. CMV, cytomegalovirus promoter; CPPT/CTS, central polypurine tract/central termination sequence element; C3, exoenzyme C3 transferase; LTR, 5′ long terminal repeat; mCMV, murine cytomegalovirus promoter; RRE, reverse response element; TAA, termination codon; WPRE, woodchuck hepatitis virus posttranscriptional regulatory element; ZsGreen, Zoanthus sp. green fluorescent protein; ψ, packaging signal.

Recombinant lentivirus vectors, expressing either green fluorescent protein (GFP) alone (LV-GFP, 5 × 10^8^ transducing units [TU]/mL) or GFP and C3 together (LV-C3-GFP, 8 × 10^8^ TU/mL), were prepared by the Beijing LKL Gene Company (Beijing, China). Lentivirus vector expressed C3 driven by a cytomegalovirus (CMV) promoter and GFP via a murine cytomegalovirus (mCMV) promoter (Fig. 1A). Briefly, the *C3* gene (657 bp) with restriction site and pLVX-mCMV-ZsGreen plasmid were digested with *Eco*RI/*Bam*HI (New England Biolabs, Ipswich, MA, USA) and linked to each other. Then the ligation products were transformed into *Escherichia coli* JM109 (Beijing LKL gene CO., Beijing, China) for further replication. The packing cell line, 293T cells (Beijing LKL gene CO.), was cotransfected with the recombinant transfer vector plasmid and lentiviral packaging vector plasmid for generation of lentiviral vectors. The viral packing supernatant was collected and centrifuged to obtain lentivirus particles. A 0.45-μm PVDF membrane filter (Merck Millipore, Billerica, MA, USA) provided sterilization, and the viral titer was evaluated by transducing HEK 293 cells, using a limiting dilution assay.

### Cell Culture

Primary HTM cells (ScienCell Research Laboratories, Inc., Carlsbad, CA, USA; see http://www.sciencellonline.com for details) were cultured in TM Cell Medium (TMCM; ScienCell Research Laboratories) consisting of basal medium, 2% fetal bovine serum, 1% TM cell growth supplement (TMCGS) and 1% penicillin/streptomycin solution, at 37°C in an atmosphere of 5% CO_2_.^7,8^ The medium was changed every other day until the cells were approximately 90% confluent. Subsequently, cells were passed sequentially in a 1:4 ratio and maintained in the same medium. Cells of passages 4 to 5 were used in the experiments.

In a 24-well plate, HTM cells (5 × 10^3^ cells) were transduced with LV-C3-GFP or LV-GFP at multiplicities of infection (MOIs) of 80, 160, 400, 640, 800, and 1130. MOI was determined by simply dividing the number of viral particles added (mL added × TU/mL) by the number of cells added per well. The optimal MOIs were evaluated through detecting the expression of GFP and changes in morphology by using an IX71-Olympus inverted microscope with Nomarski optics (IX71; Olympus, Tokyo, Japan).

### Actin Labeling

In a 6-well plate, primary HTM cells were cultured to 100% confluence (4 × 10^5^ cells) on coverslips precoated with poly-L-lysine for 1 week, at which time they exhibited a stable monolayer endothelial-like morphology. They were then transduced with LV-C3-GFP (MOI = 20 and 40) or LV-GFP (MOI = 20). Two days post treatment, the cells were washed with 1× DPBS (Dulbecco's phosphate-buffered saline), fixed with 4% paraformaldehyde (Sigma-Aldrich Corp., St. Louis, MO, USA) for 10 minutes, and then permeabilized with 0.5% Triton X-100 (Sigma-Aldrich Corp.) for 4 minutes at room temperature. The cells were blocked with 1% bovine serum albumin (Sigma-Aldrich Corp.) for 20 minutes. Rhodamine-phalloidin (Thermo Fisher Scientific, Waltham, MA, USA) was used for fluorescent labeling of actin. Nuclei were counterstained with Fluoroshield containing 4′,6-diamidino-2-phenylindole (Sigma-Aldrich Corp.). Images were captured by using a ZEISS ObserverZ1 fluorescence microscope equipped with a CCD camera (Carl Zeiss, Dublin, CA, USA). The percentage of cells with actin disruption was calculated as previously described.^9^

### Animals

All animals were maintained and handled in accordance with the Association for Research in Vision and Ophthalmology (ARVO) Statement for the Use of Animals in Ophthalmic and Vision Research. This study was approved by the Institutional Animal Care and Use Committee of Sichuan Academy of Medical Sciences. Experiments were performed on male Sprague-Dawley rats weighing 175 to 200 g, purchased from Vital River Laboratory Animal Technology Company (Beijing, China). All rats were housed in a room that remained at 22°C, 55% humidity, and 12-hour cycle lighting.

### Viral Delivery to the Anterior Segment

Rats were anesthetized with 10% chloral hydrate (0.4 mL/100 g body weight; Sigma-Aldrich Corp.) given intraperitoneally. Lentivirus suspension was delivered to the anterior chamber by using a Hamilton glass syringe with a 26s-gauge needle (10 μL volume, cemented needle, point style 2, needle length of 51 mm, needle outer diameter of 0.474 mm; Hamilton Corp., Reno, NV, USA). The corneas were anesthetized with 1 drop of 0.5% proparacaine hydrochloride (Alcaine; Alcon Laboratories, Inc., Fort Worth, TX, USA). All intracameral injections were monitored by direct visualization through an ophthalmic surgical microscope (Leica AG, Heerbrugg, Switzerland). The needle was inserted bevel-up through the peripheral cornea, and the length of the corneal puncture tunnel was approximately 1.0 to 1.5 mm. To allow for the subsequent injection volume and prevent leaking caused by injection-induced immediate elevation of IOP, a small amount of aqueous humor was released by lightly pressing the needle against the lower lip of the opening at the injection site. The anterior chamber shallowed but did not flatten upon release of aqueous humor, and it reformed immediately upon injection. 4 × 10^6^ TU (in 5–8 μL volume) of either LV-C3-GFP or LV-GFP was intracamerally injected into contralateral eyes of each animal. The needle was left in the self-sealing entry site for 1 minute and then withdrawn quickly to prevent or minimize leaking. There might have been occasional iridial-needle touch upon releasing aqueous humor, but there was no iridial or lenticular corneal contact. The upturn at the start of the bevel creates a “heel” that protects the iris from becoming impaled on the needle tip as the chamber shallows. The outer diameter of the needle is 474 μm, the central axial depth of the rat anterior chamber is ∼1.0 mm. Ofloxacin Ophthalmic Ointment (Santen, Inc., Tokyo, Japan) was topically administered to prevent ocular infection.

### Clinical Examination of the Rat Eye

To check the GFP expression, rat eyes were first set up under the bright field image of a Micron III Retinal Imaging Microscope (Phoenix Research Laboratories, Pleasanton, CA, USA) to assure that the eye was in the correct position. The anterior segments were thus also examined before looking for GFP expression at 8, 16, 21, 35, and 48 days post injection. A slit lamp biomicroscope (S350; Shanghai MediWorks Precision Instruments Co., Ltd., Hangzhou, China) with a camera attachment (EOS 600D; Canon, Inc., Tokyo, Japan) was also sometimes used to examine the anterior segment if the anesthesia permitted. The sensitivity of these two instruments was similar for observing anterior segment details. Since the rat must be anesthetized at least seven times during the entire course of the experimental timeline, the anesthesia was kept as short and “light” as possible, and no anesthesia was performed shortly after the injection.

### Intraocular Pressure Measurement

IOP readings of the conscious rats were measured by a masked investigator using the TonoLab rebound tonometer (Icare, Finland oy, Espoo, Finland) according to the manufacturer's recommended procedures. At the beginning of the experiment, rats were gently restrained by hand and trained to acclimate to the measurement procedure until stable readings were consistently achieved.^10^ Measurements were taken at the same time of the day between 9 and 11 AM on day 0 (before injection), and once daily during the first week, and then once weekly for the follow-up period until the end of the experimental timeline.

### In Vivo GFP Expression in Rats

The fluorescent image system of a Micron III Retinal Imaging Microscope was used to evaluate GFP expression in rat eyes. Rats were anesthetized with an intraperitoneal injection of 10% chloral hydrate and placed on the microscope platform. The areas of anterior segment were exposed gently by using a blunt tweezer. Image focusing was achieved by adjusting the platform. Rat position and angle relative to the microscope were altered for image acquisitions of the different quadrants of the anterior segment. After adjusting the brightness (maximum exciting light, 17 Gain and 4 frames per second for GFP detection), phase contrast and fluorescence images were captured, and analyzed by Image-Pro Plus software (Version 6.0; Media Cybernetics, Rockville, MD, USA). If the fluorescence continually remained visible in a quadrant, the fluorescent intensities at all time points were quantified by integrated optical density (IOD; Area × Average Density) and charted with Microsoft Office Excel 2016 software (Version 1805; Microsoft, Inc., Redmond, WA, USA).

### Ex Vivo Fluorescence Microscopy

Rat ocular globes were enucleated immediately after euthanization and immersed in fresh 4% paraformaldehyde in PBS overnight. Each globe was then bisected at the equator, the lens was removed, and wedge-shaped specimens containing the anterior chamber angle region with the TM were fixed for an additional hour. Specimens were then washed in 30% sucrose (4°C, overnight), embedded in OCT (Sakura Finetek USA, Inc., Torrance, CA, USA), and then frozen in liquid nitrogen. Meridional 10-μm sections were mounted on a microscope slide (Citotest Labware Manufacturing Co., Ltd., Nanjing, China) and GFP was visualized with a Leica DM4 microscope (DM400B; Leica, Wetzlar, Germany).

### Statistics

SPSS 18 software (IBM-SPSS, Chicago, IL, USA) was used for all statistics. Paired Student's *t*-test was used for the IOP comparison between the LV-C3-GFP and LV-GFP groups at the same time point after intracameral injection, and between the pretreatment and each time point after injection in the LV-GFP or LV-C3-GFP groups. One-way ANOVA was used to compare the differences of actin disruption among the three groups (including medium-only–treated control, LV-C3-GFP and LV-GFP groups). The variance was not homogeneous, so Tamhane's T2 post hoc test was used. *P* value of < 0.05 was regarded as statistically significant. Data are presented as mean ± SEM.

## Results

### Effects of C3 on HTM Cell Morphology and Actin Stress Fibers

Under our study and observation conditions, control HTM cells showed a dark polygonal shape with ruffled borders and did not show green fluorescence (Fig. 1B). Compared to the controls, HTM cells transduced with LV-C3-GFP appeared to be either elongated or rounded up at 24 hours after exposure, and the morphologic changes lasted for more than 6 days (end of observation, Fig. 1B). The number of cells showing altered morphology increased with higher MOI values, and the cells displayed cytotoxicity at MOI of 1130 (volume = 7 μL, data not shown).

**Figure 2:**
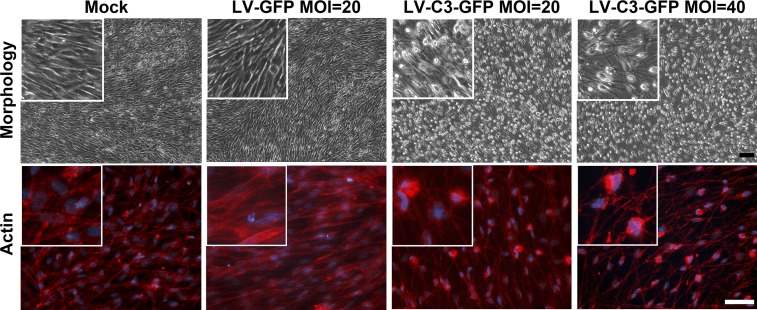
Actin organization in confluent 6-well plate monolayers of HTM cells 48 hours after transduction with lentivirus vectors. Actin was labeled with rhodamine-phalloidin (red fluorescence) and cell nuclei were labeled by DAPI (blue). Compared to LV-GFP–treated (MOI = 20) and medium-only–treated (mock) HTM cells, significant cell rounding and actin reorganization (enlarged inset images) were found in the cells receiving LV-C3-GFP (MOIs = 20 or 40). Black scale bar: 100 μm. White scale bar: 50 μm. The percentage of cells in each condition exhibiting actin disruption is shown in Table 1.

Since the C3-induced morphologic changes in HTM cells were already obvious at MOI of 80, lower MOIs of 20 and 40 were chosen for the actin-labeling experiment (volume = 10 μL and 20 μL). LV-GFP–treated and medium-only–treated cells served as viral-only and negative controls, respectively. HTM cells formed an endothelial-like monolayer with extensive intercellular contacts. As shown in Figure 2, widened intercellular spaces were found in the LV-C3-GFP–treated cells, but were not detected in the controls. There was a loss of stress fibers in cells treated with LV-C3-GFP at MOI of 20 and 40. Additionally, quantitatively significant differences in actin cytoskeleton disruption (cell rounding) were detected among the three groups, that is, LV-C3-GFP at MOI of 20, LV-GFP at MOI of 20 (volume = 16 μL), and the medium-only–treated control (Table 1). Group assignments and summary of transducing conditions are described in Table 2.

**Table 1:**
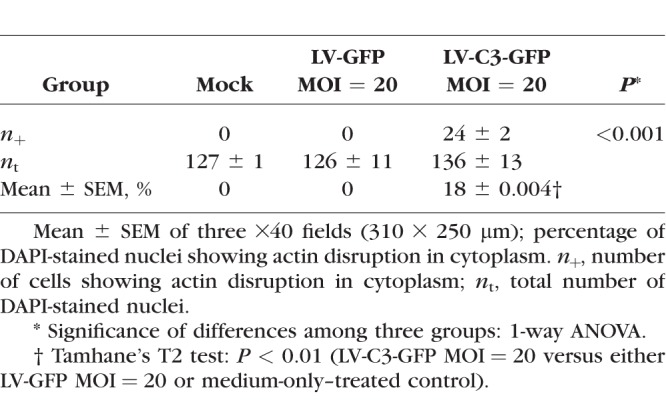
Percentage of Cells With Actin Disruption

**Table 2:**
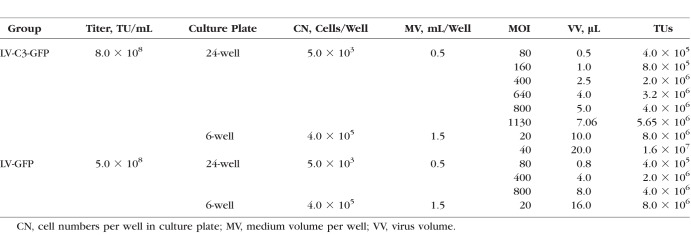
Group Assignments and Summary of Transduction for Cell Culture Study

### GFP Expression in the Anterior Segment of Rats

**Figure 3:**
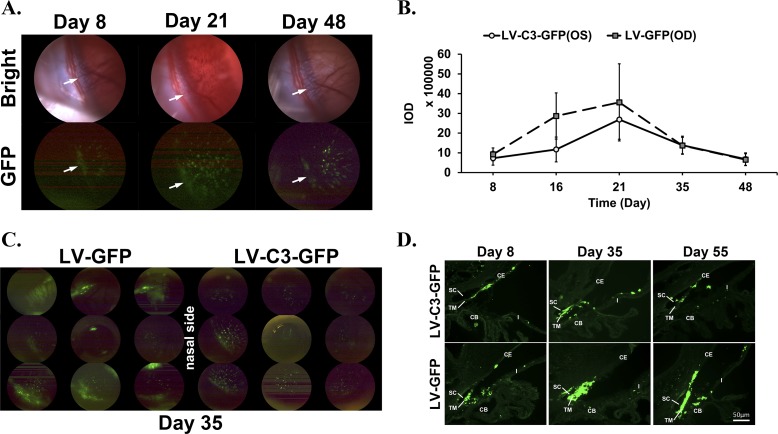
GFP expression in the anterior segment of rats. (A, C) In vivo light and fluorescence images captured by Micron III Retinal Imaging Microscope system. (D) Fluorescence images of postmortem rat ocular tissues obtained by Leica DM4 microscope. (A) GFP expression of nasal quadrant in LV-C3-GFP–injected eye of rat, at different time points. Arrows indicate fluorescence in the region of the TM. (B) IOD in nasal quadrant of rat eyes in the contralateral LV-C3-GFP and LV-GFP groups (n = 3). The peaks of GFP expression appeared on day 21. The error bars represent SEM. (C) GFP expression of all quadrants in LV-C3-GFP–injected and LV-GFP–injected rat eyes 35 days after injection. (D) Lentivirus transduction of different tissues including TM, ciliary body (CB), iris (I), and corneal endothelium (CE). SC, Schlemm's canal. Scale bar: 50 μm.

Expression of GFP after intracameral injection of LV-GFP or LV-C3-GFP was evaluated by two methods: in vivo examination and examination of postmortem ocular tissues. The rats' eyes injected intracamerally with 4 × 10^6^ TU LV-C3-GFP showed positive fluorescence (fluorescent spots and/or confluent fluorescence) in the anterior chamber angle, and in particular, the region of the TM (Fig. 3A). This fluorescence was detected first at 8 days (*n* = 14; 7 of 14 injected eyes showed fluorescence in the angle after LV-C3-GFP injection) and was intense at 16 days (*n* = 10; 8 of 10 injected eyes), 21 days (*n* = 8; 7 of 8 injected eyes), and 35 days (*n* = 7; 4 of 7 injected eyes), and faded at 48 days (*n* = 6; 3 of 6 injected eyes) (Fig. 3A; Table 3). After injection of 4 × 10^6^ TU LV-GFP, fluorescence in the angle also was detected at 8 days (*n* = 14; 8 of 14 injected eyes) and was brightest at 16 days (*n* = 10; 7 of 10 injected eyes), 21 days (*n* = 8; 5 of 8 injected eyes), and 35 days (*n* = 7; 4 of 7 injected eyes), and again weakened at 48 days (*n* = 6; 3 of 6 injected eyes) (Table 3). There were three eyes in each of the LV-C3-GFP and LV-GFP groups that still showed green fluorescence in the nasal anterior chamber angle at euthanasia at day 48. The intensity of fluorescence was quantified as IOD and charted as Figure 3B. The peak intensity of fluorescence appeared at day 21 after the injection. Although predominantly seen in the nasal and inferior regions of the angle, GFP expression was also observed in other regions, and readily visualized in vivo as a green fluorescent ring along the limbus (Fig. 3C).

**Table 3:**
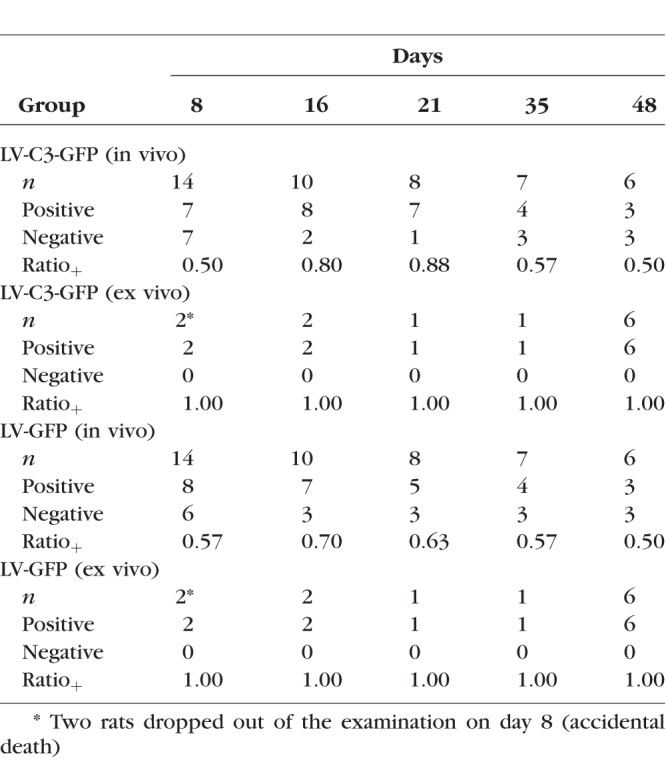
Summary of GFP Expression in the Anterior Chamber Angle of Rats After Injections

Histologically (Fig. 3D), GFP expression was visualized at 8, 16, 21, 35, and 55 days after vector injection, and the TM displayed a high level of GFP, indicating relatively specific transduction. Patches of weaker GFP expression were also visualized in the corneal endothelium, ciliary muscle, and iris endothelium.

GFP expression in the anterior chamber angle of rats was assessed by Micron III microscopy in vivo and fluorescence microscopy ex vivo at each time point. Fourteen lentivirus-injected rats were studied. When GFP expression (fluorescent spots and/or confluent fluorescence) was detected in the anterior chamber angle, the rat was counted as “positive”; “*n*” indicates the total number of rat eyes. Ratio_+_ indicates the number of eyes with GFP expression compared to total number of eyes.

### IOP Change in Rats Transduced With Lentiviral Vectors

**Figure 4:**
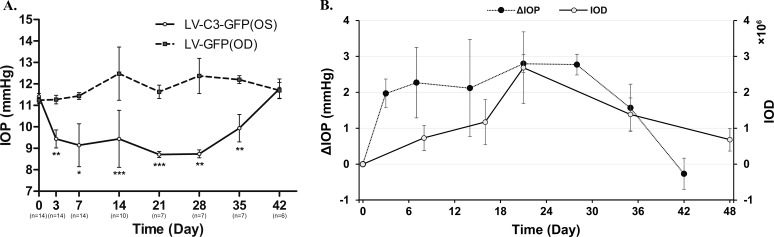
Time course of IOP response and its correlation with fluorescence intensity following LV-C3-GFP and LV-GFP transduction. (A) IOP values between LV-C3-GFP–treated eyes (4 × 106 TU) and LV-GFP–injected eyes (4 × 106 TU) were significantly different by the 2-tailed paired Student's t-test (*P < 0.05; **P < 0.01; ***P < 0.001). (B) ΔIOP indicates the difference between IOP before and after LV-C3-GFP transduction. IOD indicates the fluorescence intensity in the nasal quadrant of LV-C3-GFP–injected eyes. Error bars represent SEM.

Results of the effect of LV-C3-GFP intracameral injection on live rat IOP are shown in Figure 4. The pretreatment IOP was 11.24 ± 0.14 mm Hg (mean ± SEM, *n* = 14) and 11.41 ± 0.15 mm Hg in LV-GFP–treated and LV-C3-GFP–treated eyes, respectively, and there was no statistical difference in the baseline values of IOP between the two groups (*P* > 0.05). The IOP in the LV-GFP–treated eyes did not show significant change, compared to the pretreatment IOP (*P* > 0.05) over the course of the experiment. LV-C3-GFP (4 × 10^6^ TU) significantly lowered IOP in rats from 3 to 42 days after injection, when compared to the contralateral LV-GFP–injected eyes (*P* < 0.05), with an initial difference of 1.83 ± 0.47 mm Hg on day 3 post injection (*n* = 14; *P* < 0.01) and a maximal difference of 3.63 ± 0.83 mm Hg on day 28 (*n* = 7; *P* < 0.01). When compared to pretreatment IOP, there was a significant decrease in IOP in the LV-C3-GFP–treated eyes from 3 to 42 days (*P* < 0.05), with an initial difference of 1.97 ± 0.40 mm Hg on day 3 post injection (*n* = 14; *P* < 0.001) and a maximal decrease of 2.80 ± 0.25 mm Hg on day 21 (*n* = 7; *P* < 0.001). The IOP changes loosely approximated, but were not highly concordant with, the time-dependent expression changes of GFP (Fig. 4B). Further, minimal expression of the *C3* gene, if reflected by GFP expression, produced near-maximal IOP reduction, while maximal gene expression had little further effect on IOP.

### Monitoring Inflammation in the Rat Anterior Segment After Injection

**Figure 5:**
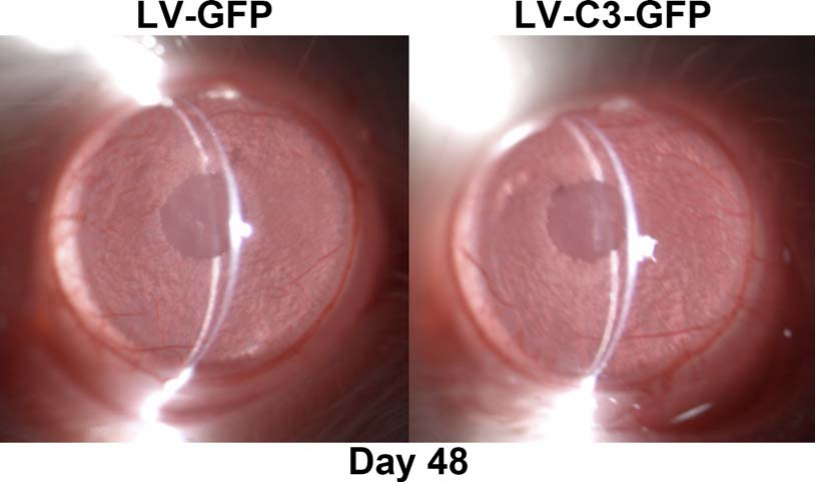
Slit-lamp biomicroscopy images obtained at day 48 after lentiviral vector injection. The conjunctiva, cornea, iris, and anterior segment of injected eyes were clear without congestion or other abnormalities.

No surgical complications, such as hyphema or cataract, occurred in the study. Rat eyes injected with LV-C3-GFP showed neither lens nor corneal opacity, or any signs of inflammation such as exudation, conjunctival or iridial hyperemia, corneal edema or anterior chamber cells or flare at any time points during the experiment, and were indistinguishable from the LV-GFP–injected eye (Fig. 5).

## Discussion

We have reported previously that adenovirus-mediated C3 expression significantly induces morphologic changes in HTM cells and increases outflow facility in organ-cultured monkey anterior segments.^6^ In this study, we confirmed that lentivirus-mediated C3 expression induced cell rounding and actin reorganization in HTM cells, and report, for the first time to the best of our knowledge, the IOP-lowering effect of lentiviral vector containing the *C3* gene in live rats.

The Rho/ROCK pathway plays a crucial role in IOP modulation of both normal and glaucomatous eyes. Many studies have demonstrated that ROCK inhibitors, such as Y-27632, H-1152, and ripasudil (K-115), increase outflow facility and reduce IOP in rats, rabbits, monkeys, and human patients.^1–4,11,12^ In addition, RhoA-siRNA effectively reduces IOP in mice by inhibiting RhoA,^13^ but this inhibition is generally accompanied by a massively increased RhoB expression, which partially compensates for the cellular functions of RhoA.^14^ C3, a 24-kDa single-chain protein from *Clostridium botulinum*,^15^ avoids this compensation by inhibiting all Rho isomers, including RhoA, RhoB, and RhoC. In our previous study, an adenoviral vector has been used to deliver C3 into the anterior chamber of organ-cultured monkey anterior segments and significantly increases outflow facility for at least 6 days. In a similar study, self-complementary adeno-associated virus serotype 2 carrying the mutated RhoA complementary DNA (scAAV2.dnRhoA) prevents elevation of nocturnal IOP in rats for at least 4 weeks.^16^ In the current study, lentivirus-mediated delivery of C3 also induced IOP lowering for more than 40 days in rat eyes. These studies indicate that IOP lowering can be achieved by inhibiting the Rho/ROCK pathway, and that expressing the *C3* gene in the TM may be an effective approach for glaucoma therapy.

We chose lentivirus as a vector for C3 transduction among various viral vectors after comparing their characteristics. Adenovirus (AdV), adeno-associated virus (AAV), herpes simplex virus (HSV), type C-retrovirus, and lentivirus are the commonly used viral vectors for ocular gene therapy trials.^17^ AdV and HSV often show different degrees of immunogenicity and limited expression durations.^18,19^ The type C-retrovirus cannot be used to transduce nondividing cells^20^ and appears to be oncogenic.^21^ The AAV vectors usually show lower immunogenicity and persistent expression^22,23^ and have been used in inherited disease studies, especially retinal and optic nerve diseases.^24,25^ The transduction efficiency of conventional AAVs in TM seems very low because it is hard for AAVs to form the double-stranded DNA by themselves.^26^ The scAAVs overcome this problem by bypassing the required second-strand DNA synthesis, and could be transduced in TM efficiently.^27–29^ However, the size of scAAV expression cassette is very limited, and in addition, the vector appears to be capable of transducing corneal endothelium (data not shown). Lentiviruses have large packaging capacity and can be integrated genomically into nondividing and dividing cells.^30^ Lentiviral vectors have the capacity of long-term efficient transduction in TM, either ex vivo or in vivo.^31,32^ Our studies showed that the C3-expressing lentiviral vector–mediated IOP-lowering effect lasted for 42 days, and GFP expression for at least 55 days in rat eyes. Similar results^33^ are reported in cats and monkeys with the Feline immunodeficiency virus (FIV)-mediated prostaglandin F synthase and GFP expression. There are several possible explanations for the gradual loss of transgene expression. Species-specific restriction factors from the host might block lentiviral infection, or the CMV promoter could be silenced in the host.^33–36^ In addition, our study showed that the GFP expression of the LV-GFP–injected eyes was stronger than that of the LV-C3-GFP–injected eyes after same amount of virus injections, but the reason remained unclear. It might be possible that expression of one gene was more efficient than simultaneously expressing two genes.

The current study showed that approximately two-thirds of the maximal IOP lowering effect was achieved with gene expression levels barely off the baseline, as shown in Figure 4B. Even though the two events were not coincident, there was a substantial IOP effect along with substantial gene expression. The reasons for the difference between fluorescence intensity and IOP lowering have not been identified, but may be explained partly by the inherent variabilities of the two parameters. However, it was also plausible that a small increase in GFP expression (as indicated by a small change in IOD) correlated with an expression level of C3 sufficient to produce the two-thirds maximal observed IOP reduction and that further increase in C3 expression produced more modest further changes in IOP. This is supported, at least in part, by the fact that the C3 expression was associated with GFP expression both in the cultured HTM cells and the live animals.

The undiluted lentiviral vectors containing the same transducing unit in different volumes (8 μL for controls and 5 μL for C3-treated group) were used to transduce rat eyes. Compared to the baseline, the IOP in LV-GFP–treated eyes did not change significantly during the experiment (from day 3 to day 42 post injection), indicating that the “overfill” in the control eyes did not affect the results. 26s-G needles were used for intracameral injections. No obvious injection-related complications such as iridial hyperemia or hyphema were found. There might have been occasional iridial-needle touch upon release of aqueous humor, but the needle, with the tip bevel-up and upturned, remained stationary, with the heel not rubbing against the iris or the needle tip touching the corneal endothelium. The central anterior chamber depth (∼1.0 mm) was approximately twice the needle outer diameter (∼0.47 mm). There was no iris deformation, iridial or lenticular corneal contact. Nonetheless, we realize that smaller-diameter needles would provide a bigger safety margin and be easier to handle for such experiments.

We did not anesthetize the animals and examine the eyes with either the Micron III or the slit lamp shortly after the injection, believing that rest for the rats was more important. However, by looking at the experimental eyes closely with our naked eyes, no obvious abnormalities such as ocular surface exudation or anterior chamber hyphema were detected. The anterior segment was examined each time when the GFP expression was checked. No abnormalities were found either (data not shown). However, the possible presence of mild and transient inflammatory reactions caused by either the injection of the vector or its transgene expression could not be ruled out for the first week post injection.

In summary, this study demonstrated that lentiviral vector–mediated C3 expression effectively induced morphologic changes and disruption of the actin cytoskeleton in primary HTM cells, and reduced IOP in rats. Thus, a lentivirus-C3 construct might be used to achieve a relatively durable IOP-lowering effect as gene therapy for glaucoma. However, the C3 expression was lost after 42 days, and in live monkeys the prostaglandin F synthase effect was not lost until 5 months. Although GFP expression in the TM could be achieved for more than 2 years in live monkeys from either an FIV^37^ or scAAV^27^ platform, neither platform carrying a *C3* transgene has yet produced an IOP effect (PLK, unpublished data). Obviously, further studies are needed.
